# Antimicrobial Effects of *Abies alba* Essential Oil and Its Application in Food Preservation

**DOI:** 10.3390/plants14132071

**Published:** 2025-07-07

**Authors:** Milena D. Vukić, Nenad L. Vuković, Marina Radović Jakovljević, Marija S. Ristić, Miroslava Kačániová

**Affiliations:** 1 Department of Chemistry, Faculty of Science, University of Kragujevac, 34000 Kragujevac, Serbia; nvchem@yahoo.com (N.L.V.); marija.jeremic@pmf.kg.ac.rs (M.S.R.); 2 Department of Biology and Ecology, Faculty of Science, University of Kragujevac, 34000 Kragujevac, Serbia; marina.jakovljevic@pmf.kg.ac.rs; 3 Institute of Horticulture, Faculty of Horticulture and Landscape Engineering, Slovak University of Agriculture, Trieda Andreja Hlinku 2, 94976 Nitra, Slovakia; miroslava.kacaniova@gmail.com; 4 School of Medical & Health Sciences, VIZJA University, Okopowa 59, 01043 Warszawa, Poland

**Keywords:** *A. alba*, antioxidant activity, antimicrobial activity, in vitro, in situ, antibiofilm activity

## Abstract

The emergence of antimicrobial resistance and the increasing demand for a healthier lifestyle have set new goals for science and industry. In the search for new, more effective, and environmentally friendly antimicrobial agents, special attention is being paid to natural resources. In this regard, essential oils derived from plants, which are widely used in the cosmetic, food, and pharmaceutical industries, are one of the solutions. In view of the above, this study aims to investigate the biological effects of *Abies alba* essential oil (AAEO). The chemical profile of AAEO was evaluated by GC/MS analysis, which revealed a high abundance of limonene (52.2%) and α-pinene (36.2%). Antioxidant activity evaluation showed a higher potential of AAEO in scavenging ABTS radical species with an IC_50_ value of 1.18 ± 0.05 mg/mL. In vitro antimicrobial activity was determined by disc diffusion and minimum inhibitory concentration assays and showed that AAEO was more efficient in inhibiting the growth of G^+^ bacterial species. On contrary, in situ evaluations of antimicrobial effects of AAEO on different food models (strawberry, kiwi, white radish, and beetroot) resulted in more efficient suppression of G^−^ bacterial species. Although AAEO showed low effects on yeasts determined by in vitro methods, in situ investigations showed its higher potential in eradication of Candida yeast. The antibiofilm properties of the AAEO matrix were determined by means of crystal violet assay and MALDI-TOF MS Biotyper analysis against biofilm-forming *Salmonella enterica*. The analysis performed led to the conclusion that AAEO, when applied prior to biofilm formation, may contribute to the removal of planktonic cells and alter the abiotic surface, thereby reducing the suitability of *Salmonella enterica* for microbial attachment.

## 1. Introduction

Modern medicine has utilized antibiotic treatment as a crucial process in treating infectious diseases. However, the emerging problem of bacterial resistance to this type of drug is threatening its value. The fact that bacterial resistance is advancing while the rate of antibiotics development decreases makes the problem more alarming [1]. The advent of this crisis has been mostly associated with the overuse, or misuse, of antimicrobial drugs in healthcare systems and industry [2]. Bearing in mind that food safety is a crucial factor in maintaining human health, antimicrobial resistance in the food industry is a growing concern [3,4]. Furthermore, the formation of biofilms (bacterial aggregation membranes created by microorganisms) shows the potential to become a persistent source of contamination in food production [5]. The ability of microorganisms to attach, grow, and form biofilms on food matrices gives them additional resistance to the conventionally used stressor agents, leading to cross-contamination. Besides the food processing sectors, this problem affects healthcare systems, water distribution systems, industrial manufacturing, marine industries, and sanitation [6].

Namely, with the aim of responding to customers’ demands for better food quality, the industrial process applies synthetic preservatives and antibiotics [7]. Nonetheless, awareness of the side effects resulting from the use of synthetic preservatives and the development of antibiotic resistance necessitates that this branch of industry develops new strategies in food protection. Inspired by the demands of the modern era toward a healthier lifestyle and environment, plant-based antimicrobial substances have been the focus of many investigations. The use of essential oils (EOs) has emerged as an alternative, especially in this field. Besides being recognized as safe, their proven antioxidant and antimicrobial activities make these plant products an attractive choice for food preservation [8]. Moreover, as EOs represent a mixture of different volatile compounds, their mechanisms of action as antimicrobial agents are diverse, which reduces the chance of the development of microbial resistance [9].

*Abies alba* Mill. (silver fir) belongs to the genus *Abies* and is one of the most complex species of the Pinaceae family, natively distributed throughout the Northern Hemisphere [10]. Besides being an important species for its environmental, economic, and social significance, silver fir has traditionally been exploited in the treatment of rheumatic arthritis and muscle pain. Novel studies on the pharmacological properties of this species have shown that *A. alba* possesses antioxidant, antimicrobial, antibiofilm, cytotoxic, antidiabetic, and anti-psoriatic effects [10,11,12].

Driven by the current global emergency, this study aimed to assess the biological potential of *A. alba* essential oil (AAEO). While AAEO obtained from different parts of the plant has been the subject of numerous studies, only a few have evaluated its ability to fight harmful bacteria that cause food spoilage and reported the efficacy of its vapor phase [11,13]. Moreover, to our knowledge, there are no previous reports on the effects of AAEO in suppressing the growth of biofilm-forming *Salmonella enterica*. Considering this, here, the chemical composition of AAEO was first determined using GC-MS analysis. Its antioxidant potential was determined by means of scavenging ABTS radical cation and DPPH radical. The antimicrobial effect was determined using various methods. The potential of AAEO to suppress the growth of G^+^ and G^−^ bacterial species and the various *Candida* yeasts was determined using MIC and disk diffusion methods. Using the vapor phase method, AAEO was further analyzed on selected food models (strawberry, kiwi, white radish, and beetroot) to evaluate its potential application as a natural preservative. In addition, the ability of AAEO to suppress the growth of biofilm-forming *Salmonella enterica* was investigated using the crystal violet assay and MALDI-TOF MS Biotyper analysis.

## 2. Results

### 2.1. Volatile Constituents’ Evaluation

The volatile profile of EO obtained from *A. alba* cones was assessed by GC/MS analysis and is presented in Table 1 as a percentage of abundance. Obtained results show that the tested essential oil was characterized by high amounts of monoterpene hydrocarbons, limonene (52.2%) and α-pinene (36.2%), making up nearly 90% of the total essential oil composition. Among them, β-pinene, representative of the same terpene class, was also identified in a significant amount of 5.3%. The rest of the six compounds present in the sample tested were detected in a total amount of 5.2%. Obtained GC/MS chromatogram of *A. alba* essential oil (Figure S1) as well as the mass spectrum of major volatiles limonene (Figure S2) and α-pinene (Figure S3) can be found in Supplementary Material.

### 2.2. Antioxidant Activity Examination

The ability of the AAEO to neutralize reactive species DPPH^•^ and ABTS^+•^, expressed as IC_50_ and TEAC values, is presented in Table 2. Generally, it can be concluded that this EO shows better effectiveness in the discoloration of ABTS radical cations compared to the DPPH radical species.

### 2.3. In Vitro Antimicrobial Activity Evaluations

As the first step, the effectiveness of AAEO in the inhibition of three G^+^ and G^−^ bacterial species and four yeasts, as well as the biofilm-forming bacteria *Salmonella enterica*, was evaluated through the disc-diffusion method. Even though this method has its limitations, its flexibility and cost make it an effective tool in screening [14]. The results obtained, presented in Table 3. indicate higher efficiency of AAEO towards G^+^ species, showing the diameter of inhibition in the range from 13.67 mm to 14.67 mm. Moreover, based on the data in Table 3, it can be concluded that AAEO has low efficiency in suppressing the growth of yeasts, showing zones of inhibition in the range from 5.67 to 8.67 mm. Furthermore, it can be noted that tested antibiotics and antimycotics were more effective in suppressing the growth of microorganisms compared to the tested AAEO.

Additionally, the broth dilutional method was employed to assess the potential of AAEO in suppressing microbial growth, and the results are presented as MIC_50_ and MIC_90_ values (Table 4). Analogously to the results of zone inhibition, AAEO was the most effective in inhibiting the growth of G^+^ bacterial species. The *Micrococcus luteus* and *Staphylococcus aureus* strains were the most susceptible to the effects of the tested EO, whose growth was suppressed to 50% at concentrations of 0.54 and 0.59 mg/mL, respectively. As for the G_¯_ strains, AAEO showed effects in the range of 2.63 to 4.49 mg/mL for MIC_50_ and from 3.19 to 5.09 mg/mL for MIC_90_, where the most susceptible to the treatment was *Escherichia coli*. The yeast strains were demonstrated to be the most resistant to the effects of AAEO. In suppression of yeasts up to 50%, AAEO showed efficiency at concentrations ranging from 3.49 to 7.62 mg/mL, where *Candida tropicalis* was the most susceptible.

### 2.4. In Situ Antimicrobial Activity Evaluations

Recent trends in the food industry, which tend to replace the use of synthetic additives in food preservation with more natural ones, have encouraged further research on the antimicrobial effects of AAEO. By employing an in situ method, the effect of the vapor phase of AAEO in the protection of the different crops (strawberries, kiwi, white radish, and beetroot) infected with the previously mentioned bacterial and yeast species was determined. From the results presented in Table 5, it can be concluded that the vapor of AAEO demonstrated the best antimicrobial effect on the strawberry model at the highest applied concentration of 500 μg/L. Applied at this concentration, AAEO was able to suppress the growth of the tested microbial species in a percentage range from 66.88% to 94.26%. The most susceptible species to its effects were found to be G^−^ *Enterobacter aerogenes*, yeast *Candida albicans*, and G^+^ *Staphylococcus aureus*. The same trend was observed in the protection of the kiwi and white radish, that is, AAEO was the most effective at the highest concentration. In the kiwi model, the tested essential oil showed the best efficiency in suppressing the growth of *Yersinia enterocolitica* (84.55%), whereas in the white radish model, similar efficiency was observed in the control of all G^−^ bacterial species (84.45–86.62%), *Candida albicans* from yeasts (85.90%), and biofilm-forming *Salmonella enterica* (85.96%). Contrary to these three food models, in beetroot, AAEO showed the highest efficiency towards the eradication of microorganisms in the lowest applied concentration (62.5 μg/L). Here, the tested EO was able to suppress the growth of microorganisms in a range from 66.96 to 77.63%, showing the lowest effectiveness compared to other investigated crops.

### 2.5. Antibiofilm Activity Evaluations

**Figure 1:**
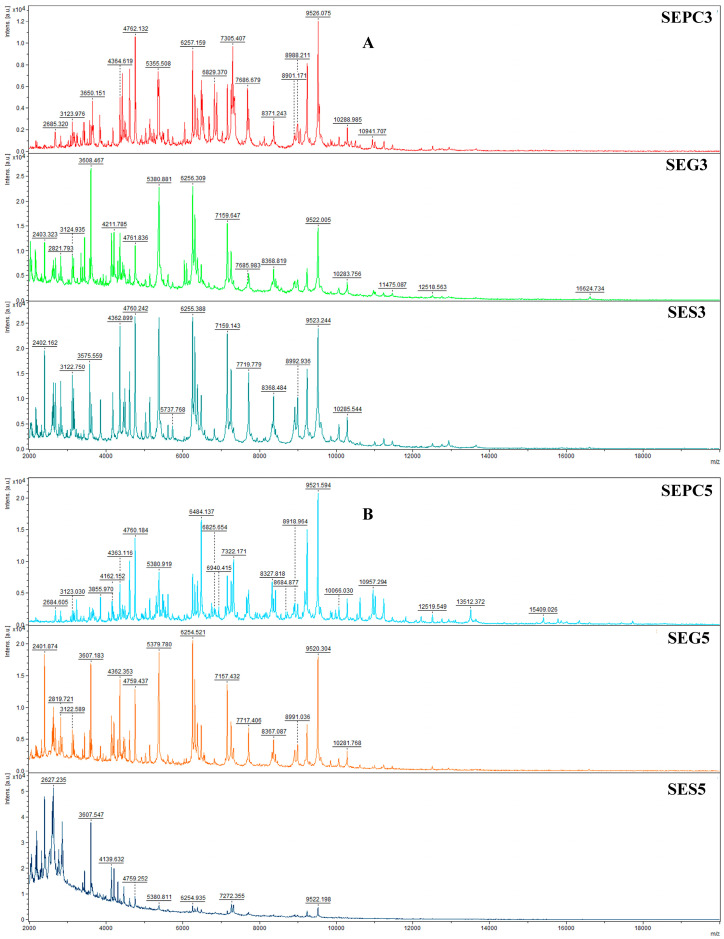

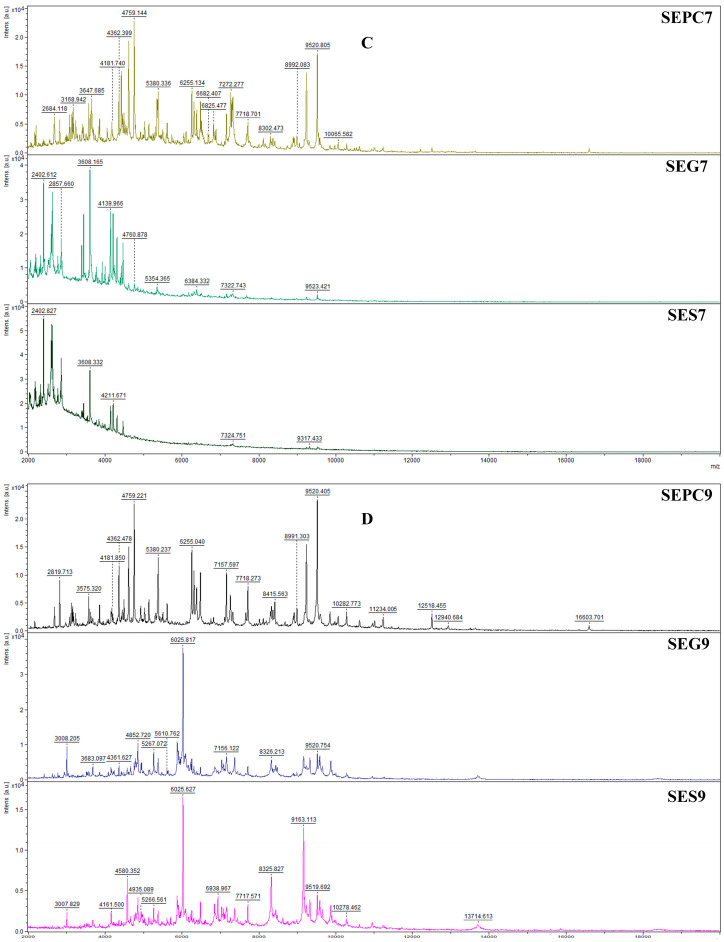

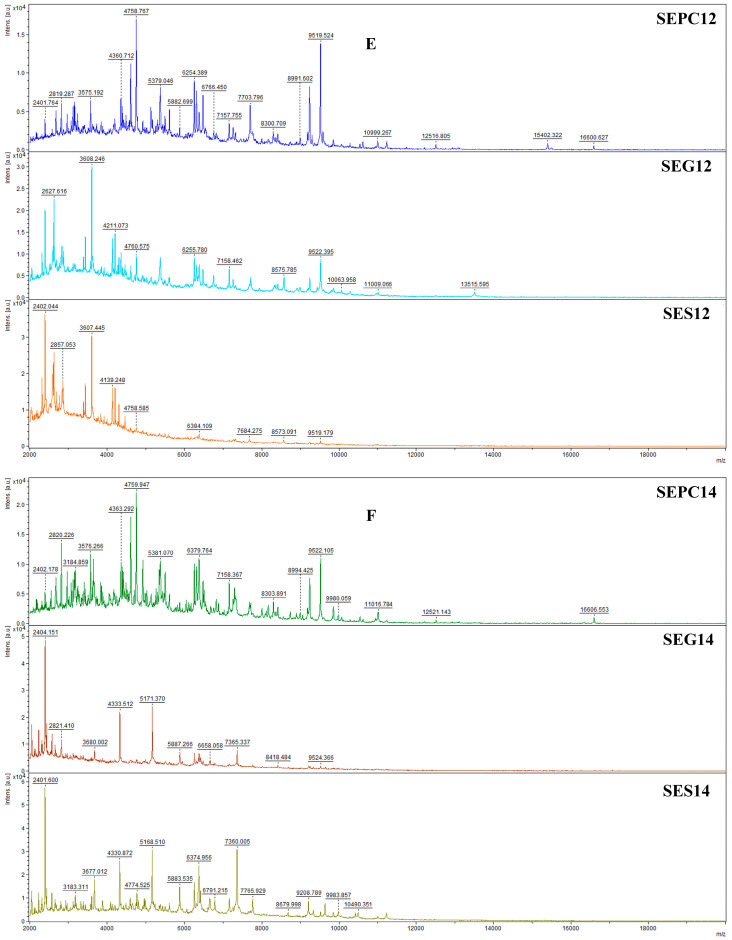
Representative MALDI-TOF mass spectra of *S. enterica*: (**A**) 3rd day; (**B**) 5th day; (**C**) 7th day; (**D**) 9th day; (**E**) 12th day; (**F**) 14th day. SE = *S. enterica*; G = glass; S = stainless steel; and PC = planktonic cells.

**Figure 2:**
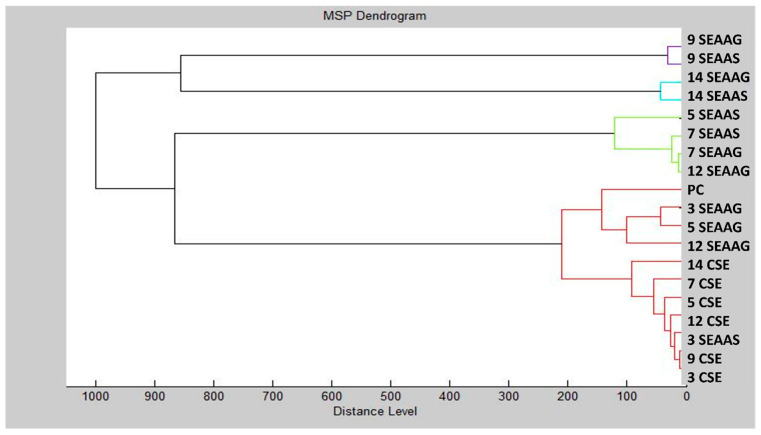
Dendrogram of *S. enterica* generated using MSPs of the planktonic cells and the control. SE = *S. enterica*; G = glass; S = stainless steel; and PC = planktonic cells.

The MALDI-TOF MS spectra illustrating the developmental stages of *Salmonella enterica* biofilms on glass and stainless steel surfaces under AAEO vapor treatment are shown in Figure 1A–F. Spectra of planktonic cells (used as a control) are also included for comparison. On the third day (SEPC3, SEG3, SES3), the spectra were highly similar between the treated and control samples, with minimal differences in peak composition and intensity. This suggests that protein expression was not yet significantly affected by the treatment at this early stage. By day 5, a divergence became apparent in the stainless steel group (SES5), where changes in peak intensity indicated the onset of antimicrobial effects. On days 7 and 9, the spectra from treated biofilms (especially SEG9 and SES9) showed clear differences from the control (SEPC9), including the appearance of new peaks (e.g., ~6025 Da, ~8300–9500 Da) and altered intensity patterns. These observations confirm that AAEO disrupted the structural and metabolic protein profile of the developing biofilm. On day 14, both SEG14 and SES14 samples exhibited an increase in peak intensity, particularly in the 2000–10,000 Da range, suggesting partial regrowth or microbial adaptation. However, the treated samples remained spectrally distinct from the control, as shown in the dendrogram analysis (Figure 2), which confirms the long-lasting effect of AAEO on biofilm protein expression. Taken together, the results indicate that AAEO vapor had a sustained impact on *S. enterica* biofilm development, with the most pronounced effects occurring between days 7 and 14 on both surfaces.

## 3. Discussion

The chemical composition of essential oils serves as their unique fingerprint, allowing the identification and characterization of their origin and potential bioactivity. Accordingly, the production of biologically active compounds in these blends depends on many factors, and the determination of the qualitative and quantitative composition of an EO can provide valuable information about its potential application. In agreement with our study, literature data show a high proportion of monoterpene hydrocarbons in EOs derived from all plant organs of AAEO [10]. In addition to this class of compounds, sesquiterpene hydrocarbons and a small amount of oxygenated terpenes (monoterpenes and sesquiterpenes) were also detected. Considering that the EO tested here indicates the presence of verbenone (monoterpene ketone) and δ-cadinene (sesquiterpene hydrocarbon), our study confirms previous results [10,11]. Regarding the major compounds, the literature provides extensive data. The production of bioactive compounds of *Abies alba* essential oils varies depending on the plant part, geographical origin, and extraction method. Thus, the essential oils extracted from needles and leaves are typically characterized by high amounts of limonene, α-pinene, β-pinene, camphene, β-phellandrene, and bornyl acetate, while the essential oils extracted from seeds have a high concentration of limonene and a moderate presence of α-pinene [10,15,16,17,18,19]. The EOs obtained from twigs and branches can be categorized into two chemotypes: the first is rich in limonene, camphene, and α-pinene, while the second is dominated by camphene, α-pinene, limonene, β-phellandrene, and β-pinene, together with minor constituents such as myrcene, tricyclene, and α-terpineol [17]. Although the results on the AAEO extracted from cones are modest, some previously published data indicate higher amounts of α-pinene compared to limonene [19]. Contrary to this, our study showed higher abundance of monoterpene hydrocarbons limonene (52.2%), compared to α-pinene (36.2%). A study provided by Garzoli et al. in 2021 [11] showed similar amounts of these two monoterpene hydrocarbons in the tested sample of AAEO (32.5% of limonene and 30.8% of α-pinene).

Evaluation of the antioxidant properties of essential oils may indicate their potential in food preservation by preventing chemical spoilage of food. Previous research indicates that AAEO shows diverse antioxidant effects. When evaluating the inhibition of the DPPH radical by AAEO, Garzoli et al., in 2021 [11], determined an IC50 value of 7.84 ± 1.70 µg/mL for AAEO from needles, Marjanovic-Balaban et al., in 2020 [20], showed that two samples of AAEO obtained by two different methods from the twigs with cones had EC50 values of 32.0 ± 0.03 mg/mL and 29.93 ± 0.08 mg/mL, while the results of Wajs-Bonikowska et al., in 2015 [19], reported values of 0.215 ± 0.014 and 0.591 ± 0.039, expressed as mm Trolox equivalent per liter, for AAEO obtained from seeds and cones, respectively. These differences can be attributed to the chemical composition of the essential oils tested, which depends, among other things, on the plant material used to extract the essential oil, the method used to isolate the essential oil, and the method used to determine DPPH neutralization, as well as the way in which the results are presented. Given the uniqueness of each sample, our next step was to determine the antioxidant potential of the AAEO studied in this work. Investigation of the antioxidant effects showed the superiority of the tested AAEO in scavenging ABTS radical cations compared to DPPH radicals. This result aligns with a previous study which demonstrated the superiority of ABTS^+•^ neutralization compared to the neutralization of DPPH^•^, especially in the presence of antioxidants containing high-pigmented, lipophilic, and hydrophilic compounds [21]. These observations were also confirmed in our previous investigations [22]. Contrary to the presented results, Ancuceanu et al., in 2023 [10], indicated limonene as a stronger inhibitor of DPPH^•^ and a mild inhibitor of ABTS^+•^. The discrepancies can be attributed to the complexity of the matrix represented by essential oils and the synergistic effects of the compounds present.

Based on the presented results of volatile identification, the general conclusion is that the tested essential oil is a rich source of monoterpene hydrocarbons, with limonene (52.2%) and α-pinene (36.2%) being the major components. The rationale of this study was based on the previously published data, which characterizes these cyclic monoterpenes as potent growth inhibitors of a wide spectrum of microbial species [1,23,24]. Despite the fact that some previous results indicate that EOs obtained from *A. alba* species are devoid of or show low antimicrobial effects, here, we have demonstrated at least moderate outcomes [10,19,25,26]. As the overall conclusion, the observed differences in the results may be affected by factors such as seasonal variation, soil conditions, region of cultivation, plant parts used, and extraction methods. These play a crucial role in the levels and types of secondary metabolites produced in plants, which may determine the strength of antimicrobial activity [26,27,28]. The significant efficacy of AAEO against G^+^ bacteria alongside weaker effects on G^−^ strains could be the result of the unique structure of their outer membrane, which acts as a barrier to EO compounds. G^−^ bacteria are known to be less sensitive to EOs due to their hydrophilic outer membrane, which limits the diffusion of active compounds [29]. Recent research by the authors of reference [30] qualified limonene as a broad-spectrum antibacterial agent with multiple targets. However, it should be noted that compared to the pure compounds, synergistic and antagonistic effects expressed in mixtures such as EOs have a strong influence on their expressed biological activities. In the study provided by the authors of reference [31], it was found that the antibacterial effect of limonene could be improved in EO, compared to its effects as a single compound. Furthermore, previous studies show that α-pinene can enhance the permeability of bacterial membranes in addition to inducing an intracellular accumulation of antibiotics due to the inhibition of antimicrobial efflux systems [32,33].

It should be borne in mind that food safety is a general, worldwide problem. When it comes to food safety, most problems occur with fresh fruit and vegetables, which make up the largest part of the food cycle. As the trend towards healthy eating increases, so does the pressure on the environment from intensive farming and post-harvest handling. After harvest, fresh horticultural produce is exposed to numerous physiological influences that can lead to rot and disease [34]. Stress during processing can affect quality and lead to significant post-harvest losses, especially in developing countries. Much of this damage remains hidden until the end of the supply chain, driving up costs due to waste and spoilage [35]. In addition, consumers have recently become more inclined towards healthier lifestyles. As a result, there is growing interest in safer, environmentally friendly alternatives to synthetic chemicals, such as the use of EOs to combat putrefaction and maintain product quality. One of the advantages of EOs as a compatible alternative is that these volatile liquids have been recognized as safe by the US FDA (Food and Drug Administration) and the EPA (Environmental Protection Agency) [36]. Furthermore, its wide use in various industries (food, cosmetic, and pharmaceutical) underlines its potential as an effective and sustainable solution to mitigate post-harvest decay. Although the antimicrobial efficacy of EOs has been widely studied in direct contact, new research has shown that these volatile liquids may have greater potential in the gas or vapor phase [37]. In this regard, our study underscores the overall versatility of AAEO vapor as an effective antimicrobial agent against various pathogens affecting different crop types. However, from the obtained results, it can be concluded that G^−^ species were more sensitive to the vapor of AAEO in contrast to the results obtained by the MIC method, including direct contact treatment. In accordance with the results obtained here, reference [11] demonstrated a greater efficacy of AAEO in the vapor phase compared to its liquid phase against various bacterial strains tested in vitro. In addition, the study presented by the authors of reference [13] characterized AAEO as a promising natural agent for food storage (including bakery products and vegetables) against fungal species. In summary, these results may suggest that AAEO can be further utilized for natural food preservation strategies, where future research should focus on elucidating the mechanisms behind crop-specific efficacy variations and evaluating sensory effects to ensure consumer acceptance.

According to the WHO (2018), about one in ten people fall ill from foodborne diseases every year, with Salmonella infections being one of the leading causes of diarrheal disease worldwide [38]. Our study has shown that AAEO has a small effect on the eradication of *S. enterica* biofilm in the crystal violet test (CV). However, at the highest concentration applied, AAEO vapor effectively suppressed the growth of biofilm-forming *S. enterica* on strawberries, kiwi, and white radish. In contrast, in the beetroot model, the lowest concentration applied showed the best antimicrobial effects. These results can also lead to the conclusion that the vapor phase of the tested EO has higher antibiofilm effects. Additional studies conducted using MALDI-TOF mass spectrometry analysis imply that AAEO, when applied prior to biofilm development, could help remove planktonic cells and modify the abiotic surface so that it is less favorable for cell adhesion. Additionally, obtained results suggests that higher dosages of AAEO are likely to be more effective in long-term inhibition of *S. enterica* biofilm development.

## 4. Materials and Methods

### 4.1. Reagents and Equipment

Methanol (MeOH), ethanol, hexane, ethyl acetate (EtOAc), 2,2-diphenyl-1-picrylhydrazyl (DPPH), 6-hydroxy-2,5,7,8-tetramethylchroman-2-carboxylic acid (Trolox), 2,20-azinobis (3-ethylbenzothiazoline- 6-sulfonic acid) diammonium salt (ABTS), and potassium persulfate (K_2_S_2_O_8_) were purchased from Sigma-Aldrich (Darmstadt, Germany), while Mueller–Hinton Broth (MHB), Mueller–Hinton Agar (MHA), Sabouraud Dextrose Broth (SDB), Sabouraud Dextrose Agar (SDA), cefoxitin, gentamicin antibiotics, and fluconazole as antimycotic (30 µg/disc) were acquired from Atom Scientific (Oxoid, Basingstoke, UK). For spectroscopic measurements, we used a Glomax spectrophotometer (Promega Inc., Madison, WI, USA). GC/MS analysis was conducted on an Agilent Technologies (Santa Clara, CA, USA) 6890N gas chromatograph equipped with an HP-5MS fused silica column (30 m × 0.25 mm × 0.25 μm, Agilent Technologies) and an Agilent Technologies mass-selective detector 5975B. Mass spectrometry (MALDI-TOF MS Biotyper) was used for antibiofilm activity (Brucker Daltonics, Bremen, Germany).

### 4.2. Essential Oil

The essential oil of *Abies alba* (AAEO) used in the evaluation of its chemical profile and biological activity determination studies was acquired from Hanus s.r.o. (Nitra, Slovakia). Based on the data provided by the manufacturer, tested EO was obtained by distillation of cones originating from Slovakia. Prior to use, the AAEO was stored in dark conditions at 4 °C.

### 4.3. Determination of the Chemical Profile by Employing GC/MS Analysis

For the evaluation of the exact volatile content in the EO, the sample was diluted in hexane to obtain a 10% solution. Additional parameters for GC/MS analysis were set as follows:

1. MS ion source temperature at 230 °C;
2. MS quadrupole temperature at 150 °C;
3. Split mode set with a split ratio of 40.8:1;
4. Flow rate of the carrier gas (Helium 5.0) at 1 mL/min;
5. Electron-impact mass spectrometric data (EI-MS; 70 eV) were acquired in scan mode over the m/z range 35–550.

The diluted sample was next injected in a volume of 1 μL. Analysis was carried out over 57 min, whereas the data acquisition was set to start after 3.2 min of solvent delay time. The temperature program for the oven was as follows: from 50 °C with an increase of 3 °C/min to 75 °C with a hold of 4 min, from 75 °C with an increase of 5 °C/min to 120 °C (hold for 2 min), from 120 °C with an increase of 5 °C/min to 290 °C. Additionally, with the aim of volatile identification, retention times for a standard series of *n*-alkanes (C_7_–C_35_) were obtained in the same manner, with the solvent delay time set at 2.10 min (to obtain the retention index for n-heptane). In the final step, the compounds were identified by comparing the recorded mass spectra with the mass spectral libraries (Wiley7 and NIST) and by correlating the MS data and the experimentally determined Kovats retention indices with values from the literature [39]. Semi-quantification of compounds present at concentrations greater than 0.1% was performed using MSD ChemStation software D.03.00.611 (Agilent Technologies, USA) based on the area of the corresponding GC peaks.

### 4.4. Antioxidant Activity

Antioxidant effects of the AAEO were determined by using the standard DPPH and ABTS methods described previously [40,41]. Briefly, prepared solutions of DPPH radical and ABTS radical cation (190 μL) were added to a 96-well microtiter plate, followed by the addition of 10 μL of AAEO sample (in the concentration range of 3.0 mg/mL to 0.1875 mg/mL in methanol) or the reference compound Trolox (in the concentration range of 3.0 to 0.015 mg/mL). The prepared reaction mixture was shaken at 1000 rpm for 30 min at room temperature in the dark. Finally, the absorbance was measured using a microplate reader at 515 nm and 744 nm for the DPPH and ABTS assays, respectively. Results are presented as the means of IC_50_ and TEAC values of three independent measurements reported as mean values ± standard deviation (SD) [42,43].

### 4.5. In Vitro Antimicrobial Evaluations

The antimicrobial efficiency of the AAEO was evaluated on a microbial panel from the Czech collection of microorganisms (CCM, Brno, Czech Republic). For this purpose, G^+^ bacterial species *Listeria monocytogenes* CCM 4699, *Micrococcus luteus* CCM 732, *Staphylococcus aureus* CCM 3953, G^−^ *Enterobacter aerogenes* CCM 2531, *Escherichia coli* CCM 3953, *Yersinia enterocolitica* CCM 5671, and yeasts *Candida albicans* CCM 8186, *Candida glabrata* CCM 8270, *Candida krusei* CCM 8271, and *Candida tropicalis* CCM 8223 were used. In the assessment of antibiofilm activity, biofilm-forming G^−^ bacterial species *Salmonella enterica* was extracted and sequenced from milk production. The assessment was performed by two standard methods, disc-diffusion and the broth microdilution method. Before analysis, bacterial inoculums were cultured in Mueller–Hinton Broth (MHB) for a full day at 37 °C, and yeast inoculums were cultured in Sabouraud Dextrose Broth (SDB). On the day of the experiment, the optical density of the bacterial inoculum was set at 0.5 McFarland standard.

#### 4.5.1. Disc Diffusion Method

The experiment was performed by inoculation of prepared microbial strains in a volume of 100 μL on the Mueller–Hinton Agar (MHA) for bacteria and Sabouraud Dextrose Agar (SDA) for yeasts [44]. Thereafter, blank discs (6 mm) soaked with 10 μL of AAEO were placed on the agar surface alongside the discs containing cefoxitin, gentamicin, and fluconazole as a positive control. Sterile blank discs without any added compound were used as negative controls to assess the intrinsic activity of the paper disc. After the incubation period of 24 h at 37 °C resp. 25 °C, inhibition diameters were recorded in mm as the efficiency of bacterial inhibition.

#### 4.5.2. Broth Dilution Method

Evaluation of the minimal inhibitory concentration was performed by the method described earlier by the authors of reference [44]. Briefly, freshly prepared bacterial and yeast inoculums (50 μL) were placed in the 96-well microtiter plates, followed by the addition of AAEO (concentration range of 100–0.05 mg/mL). Additionally, pure MHB with bacterial inoculum or MHB with AAEO and pure SDB with yeasts inoculum or SDB with AAEO in the same concentration range were used as controls. Once the incubation period was finished (24 h at 37 °C resp. 25 °C), plates were placed in a spectrophotometer and absorbance was recorded at 570 nm. The results of the experiment were presented as MIC values (MIC50 and MIC90).

### 4.6. In Situ Antimicrobial Evaluations

With the aim of assessing the protective effects of AAEO on the strawberries, kiwi, white radish, and beetroot as food models against different bacterial and yeast strains (used in in vitro evaluations), an in situ analysis was performed. Commercially available food substrates were cut into 0.5 mm segments, washed, and dried. Afterward, prepared slices of food models were placed in the 60 mm Petri dishes supplemented with MHA and SDA. Different concentrations of AAEO (62.5–500 µg/L in EtOAc) or pure EtOAc (as control) were next added to the filter paper and attached to the top of the Petri plates. The essential oil was applied in the vapor phase to simulate realistic food storage conditions and to prevent direct contact with the food surface, which could affect its sensory and visual properties. After, sealed plates were incubated for 7 days at 37 °C. The volume density of bacterial colonies was determined using the ImageJ tool (software version 1.8.0 from the National Institutes of Health in Bethesda, MD, USA), and the results are presented as a percentage of bacterial inhibition [44].

### 4.7. Antibiofilm Evaluations

The biofilm eradication capacity of AAEO was evaluated using two different methods, the crystal violet assay and by use of MALDI-TOF MS Biotyper for the detection of biofilm formation. For this purpose, we have used the biofilm-forming G^−^ bacteria *Salmonella enterica* isolated and sequenced from milk products. Bacterial inoculums were prepared as described above.

#### 4.7.1. Crystal Violet Assay

The efficiency of AAEO in inhibiting the formation of biofilm was assessed by the crystal violet (CV) assay based on dyeing attached cells on culture plates [45]. The experiment was performed by adding 50 μL of bacterial inoculum alongside the same volume of AAEO in different concentrations (from 100 to 0.05 mg/mL). After an incubation period of 24 h, the attached cells were washed, dried, and stained with CV (0.1%). Fifteen minutes later, the excess of dye was removed with ultrapure water and dried, after which fixed dye (crystal violet) was released with 96% ethanol. The absorbance of the released dye was measured at 570 nm, and the results are presented as MBIC (minimal biofilm inhibitory concentration).

#### 4.7.2. MALDI-TOF MS Study

Using a Bruker Daltonics MALDI-TOF MicroFlex apparatus, the effectiveness of AAEO in preventing the formation of biofilms caused by biofilm-forming G^−^ bacteria *Salmonella enterica* on glass and stainless-steel surfaces was determined [46]. In the polypropylene tubes supplemented with MHB (20 mL) and bacterial suspension (100 μL) as a control or MHB (20 mL), bacterial suspension (100 μL), and AAEO (0.1%), slides of the investigated surfaces were placed. Prepared tubes were left on a shaker (170 rpm) and incubated at 37 °C for 3, 5, 7, 9, 12, and 14 days. On each day of the experiment, from the tested surfaces, biofilms were taken by a sterile swab and analyzed on the MALDI-TOF to record spectral changes. Additionally, planktonic cells from control samples lacking AAEO were also analyzed. Spectral data were obtained with the mass-to-charge ratio set at 200–2000 in linear positive mode. Dendrograms were generated using 19 standard global spectrums (MSP) by employing the Euclidean Distance Formula.

### 4.8. Statistical Analysis

The data were expressed as mean values ± standard deviation (SD), and each assessment was conducted in triplicate. A one-way ANOVA was performed, followed by Tukey’s HSD test at a significance level of *p* ≤ 0.05 using the SPSS software package (IBM SPSS Statistics 20). Changes in absorbance between measurements were transformed into a set of binary values using the measured absorbances obtained before and after the experiment. Specific concentrations were assigned to these values based on the following formula, developed for this experiment: if absorbance values were equal to or lower than 0.01 (indicating an inhibitory effect), the binary system’s numbers were set to 1; if absorbance values were higher than 0.01 (indicating no effect or a stimulant impact), the binary system’s numbers were set to 0. Finally, the JMP Pro 17.0 software program (SAS Institute, Cary, NC, USA) was used for graphic elaborations.

## 5. Conclusions

This study aimed to exploit the EO of *A. alba* with the aim of investigating its use as an antimicrobial in food preservation. Our results on the chemical composition of AAEO indicated a high content of biologically attractive limonene and α-pinene. In addition, the high antioxidant potential of AAEO towards inhibition of DPPH radical and ABTS radical cation was determined. These results led to further investigation of the comprehensive antimicrobial potential of AAEO. The in vitro investigation showed mild effects of the tested EO in inhibition of bacterial and yeast strains, with G^+^ strains being the most sensitive. In contrast, in situ investigations conducted with the goal of exploring the crop-protective nature of AAEO vapor showed its higher potential to suppress bacterial and yeast species. This study showed that AAEO has the highest effectiveness in the suppression of G^−^ species growing on strawberries, kiwi, white radish, and beetroot. Additionally, this study demonstrated the potential of AAEO to provide long-lasting disruption of the homeostasis of the *S. enterica* biofilm.

Overall, it can be concluded that the tested AAEO expresses higher antimicrobial activity in the vapor phase and may have potential as a natural food preservative and as an antibiofilm agent in the eradication of highly infectious *S. enterica*.

## Figures and Tables

**Table 1:** Chemical composition of *A. alba* EO.

No	RI (Lit.) ^a^	RI (Calc.) ^b^	Compound ^c^	% ^d^
1	939	935	α-pinene	36.2
2	954	951	camphene	tr
3	967	954	verbenene	1.4
4	979	977	β-pinene	5.3
5	990	988	β-myrcene	1.5
6	1026	1027	*o*-cymene	tr
7	1029	1033	limonene	52.2
8	1205	1205	verbenone	0.8
9	1523	1517	δ-cadinene	1.5
			total	98.9

^a^ Experimental values of retention indices on HP-5MS column. ^b^ Literature values of retention indices. ^c^ Identified compounds. ^d^ The percentage of the identified compound; tr—compounds identified in amounts less than 0.1%.

**Table 2:** Antioxidant activity of *A. alba* EO.

	IC_50_ (mg/mL)	TEAC
ABTS^+•^	1.18 ± 0.05	0.0014
DPPH^•^	2.33 ± 0.01	0.0020

Data are presented as the mean (±SD) of three repetitions.

**Table 3:** Antimicrobial and antibiofilm effects of *A. alba* obtained by using the disc-diffusion assay.

Microorganism	Inhibition Zone (mm)	
	AAEO	ATB/AMC
Gram-positive bacteria		
*Listeria monocytogenes* CCM 4699	14.67 ± 0.58 ^d^	27.67 ± 0.58 ^a,b,c^
*Micrococcus luteus* CCM 732	14.33 ± 0.58 ^d^	29.67 ± 0.58 ^d,e^
*Staphylococcus aureus* CCM 3953	13.67 ± 0.58 ^d^	28.67 ± 0.58 ^b,c,d^
Gram-negative bacteria		
*Enterobacter aerogenes* CCM 2531	9.33 ± 0.58 ^b,c^	27.33 ± 0.58 ^a,b^
*Escherichia coli* CCM 3953	10.67 ± 0.58 ^c^	30.67 ± 0.58 ^e^
*Yersinia enterocolitica* CCM 5671	8.67 ± 0.58 ^b^	29.33 ± 0.58 ^c,d,e^
Yeast		
*Candida albicans* CCM 8186	5.67 ± 0.58 ^a^	27.67 ± 0.58 ^a,b,c^
*Candida glabrata* CCM 8270	7.67 ± 0.58 ^b^	26.67 ± 0.59 ^a^
*Candida krusei* CCM 8271	8.67 ± 0.58 ^b^	27.33 ± 0.58 ^a,b^
*Candida tropicalis* CCM 8223	7.67 ± 0.58 ^b^	26.67 ± 0.58 ^a^
Biofilm-forming bacteria (BFB)		
*Salmonella enterica*	10.67 ± 0.58 ^c^	27.67 ± 0.58 ^a,b,c^

Data are the mean (±SD) of three repetitions. Different letters in each column refer to significant differences (Tukey, *p* ≤ 0.05). ATB = antibiotics, AMC = antimycotic.

**Table 4:** Antimicrobial effects of *A. alba* EO obtained by using the minimal inhibitory concentration assay expressed in mg/mL.

Microorganism	MIC_50_	MIC_90_
Gram-positive bacteria		
*Listeria monocytogenes* CCM 4699	0.67 ± 0.09 ^a^	0.77 ± 0.10 ^a^
*Micrococcus luteus* CCM 732	0.54 ± 0.06 ^a^	0.64 ± 0.06 ^a^
*Staphylococcus aureus* CCM 3953	0.59 ± 0.06 ^a^	0.63 ± 0.03 ^a^
Gram-negative bacteria		
*Enterobacter aerogenes* CCM 2531	3.07 ± 0.53 ^b,c^	4.19 ± 0.46 ^d,e^
*Escherichia coli* CCM 3953	2.63 ± 0.26 ^b^	3.19 ± 0.59 ^b,c^
*Yersinia enterocolitica* CCM 5671	4.49 ± 0.14 ^d^	5.09 ± 0.30 ^f^
Yeast		
*Candida albicans* CCM 8186	7.62 ± 0.22 ^f^	8.05 ± 0.24 ^h^
*Candida glabrata* CCM 8270	6.54 ± 0.2 ^e^	7.07 ± 0.23 ^g^
*Candida krusei* CCM 8271	4.34 ± 0.22 ^d^	4.81 ± 0.05 ^e,f^
*Candida tropicalis* CCM 8223	3.49 ± 0.29 ^c^	3.95 ± 0.05 ^c,d^
Biofilm-forming bacteria (BFB)		
*Salmonella enterica*	2.45 ± 0.10 ^b^	2.74 ± 0.27 ^b^

Data are the mean (±SD) of three repetitions. Different letters in each column refer to significant differences (Tukey, *p* ≤ 0.05).

**Table 5:** In situ analysis of the antimicrobial activity of the vapor phase of AAEO in strawberry, kiwi, white radish, and beetroot.

Food Model	Microorganisms	Percent of Inhibition of EO (μg/L)			
Strawberries		62.5	125	250	500
G+	*Listeria monocytogenes*	33.26 ± 1.14 ^b^	45.40 ± 0.55 ^b^	55.51 ± 1.49 ^a^	66.88 ± 2.01 a
	*Micrococcus luteus*	45.11 ± 2.99 ^c^	53.89 ± 2.78 ^c^	65.63 ± 1.16 ^b^	75.66 ± 1.72 ^a,b^
	*Staphylococcus aureus*	6.26 ± 1.14 ^d^	34.76 ± 2.03 ^d^	55.68 ± 2.02 ^c^	85.63 ± 0.95 ^c,d^
G−	*Enterobacter aerogenes*	35.07 ± 3.14 ^b^	54.13 ± 3.56 ^c^	74.63 ± 3.99 ^c^	94.26 ± 3.50 ^d^
	*Escherichia coli*	13.92 ± 0.55 ^a^	34.60 ± 1.89 ^a^	55.70 ± 2.66 ^a^	77.62 ± 1.79 ^b,c^
	*Yersinia enterocolitica*	34.00 ± 0.59 ^b^	45.17 ± 1.04 ^b^	55.75 ± 1.82 ^a^	73.82 ± 2.56 ^a,b^
Yeast	*Candida albicans*	29.33 ± 5.72 ^b^	45.51 ± 1.01 ^b^	66.04 ± 2.15 ^b^	86.36 ± 2.43 ^c,d^
	*Candida glabrata*	44.37 ± 2.52 ^c^	55.95 ± 0.64 ^c^	65.33 ± 2.20 ^b^	74.97 ± 3.78 ^a,b^
	*Candida krusei*	33.92 ± 1.66 ^b^	44.37 ± 1.13 ^b^	57.20 ± 2.17 ^a^	73.18 ± 6.40 ^a,b^
	*Candida tropicalis*	32.63 ± 1.27 ^b^	43.95 ± 2.02 ^b^	54.93 ± 2.17 ^a^	66.73 ± 2.90 ^a^
BFB	*Salmonella enterica*	43.81 ± 1.64 ^c^	54.10 ± 2.77 ^c^	63.78 ± 2.22 ^b^	75.60 ± 2.97 ^a,b^
Kiwi					
G^+^	*Listeria monocytogenes*	36.08 ± 1.31 ^c^	46.11 ± 0.76 ^b^	56.77 ± 1.00 ^b^	66.40 ± 1.56 ^b^
	*Micrococcus luteus*	24.85 ± 2.22 ^b^	35.37 ± 1.39 ^a^	44.33 ± 1.25 ^a^	57.14 ± 1.67 ^a^
	*Staphylococcus aureus*	43.10 ± 0.40 ^d^	55.34 ± 2.23 ^c^	63.56 ± 2.28 ^c^	73.89 ± 2.51 ^c^
G^−^	*Enterobacter aerogenes*	17.44 ± 0.58 ^a^	33.56 ± 1.06 ^a^	46.07 ± 2.62 ^a^	75.96 ± 1.22 ^c^
	*Escherichia coli*	24.60 ± 1.89 ^b^	37.63 ± 3.06 ^a^	55.71 ± 1.86 ^b^	76.58 ± 2.05 ^c^
	*Yersinia enterocolitica*	24.44 ± 1.32 ^b^	43.60 ± 2.06 ^b^	64.92 ± 3.03 ^c^	84.55 ± 1.84 ^d^
Yeast	*Candida albicans*	33.66 ± 1.01 ^c^	44.27 ± 1.23 ^b^	55.11 ± 2.08 ^b^	64.56 ± 2.94 ^b^
	*Candida glabrata*	17.14 ± 1.68 ^a^	35.63 ± 3.04 ^a^	54.07 ± 1.12 ^b^	73.39 ± 1.20 ^c^
	*Candida krusei*	43.74 ± 1.75 ^d^	58.43 ± 1.52 ^c^	64.60 ± 1.17 ^c^	76.03 ± 2.09 ^c^
	*Candida tropicalis*	33.63 ± 0.96 ^c^	47.44 ± 1.14 ^b^	55.07 ± 1.58 ^b^	66.05 ± 1.63 ^b^
BFB	*Salmonella enterica*	45.40 ± 3.50 ^d^	53.41 ± 2.92 ^c^	64.42 ± 1.09 ^c^	76.13 ± 1.98 ^c^
White radish					
G^+^	*Listeria monocytogenes*	44.29 ± 1.45 ^b^	56.29 ± 2.46 ^b^	64.02 ± 1.25 ^b^	75.71 ± 1.11 ^b^
	*Micrococcus luteus*	36.14 ± 1.66 ^a^	45.30 ± 1.84 ^a^	56.04 ± 2.32 ^a^	67.39 ± 1.80 ^a^
	*Staphylococcus aureus*	45.26 ± 1.87 ^b^	55.33 ± 2.30 ^b^	64.81 ± 0.91 ^b^	74.52 ± 2.94 ^b^
G^−^	*Enterobacter aerogenes*	56.59 ± 2.16 ^c^	64.56 ± 3.21 ^c^	75.22 ± 2.18 ^c^	84.45 ± 3.17 ^c^
	*Escherichia coli*	55.18 ± 4.80 ^c^	65.43 ± 2.24 ^c^	77.61 ± 1.23 ^c^	86.62 ± 1.12 ^c^
	*Yersinia enterocolitica*	44.19 ± 1.30 ^b^	56.60 ± 2.17 ^b^	64.74 ± 1.06 ^b^	85.32 ± 3.14 ^c^
Yeast	*Candida albicans*	55.36 ± 1.27 ^c^	65.91 ± 1.67 ^c^	76.19 ± 2.22 ^c^	85.90 ± 0.59 ^c^
	*Candida glabrata*	33.43 ± 1.20 ^a^	45.61 ± 2.31 ^a^	56.70 ± 2.04 ^a^	66.70 ± 2.04 ^a^
	*Candida krusei*	35.23 ± 2.19 ^a^	45.74 ± 1.89 ^a^	55.37 ± 2.27 ^a^	66.85 ± 1.78 ^a^
	*Candida tropicalis*	35.30 ± 1.70 ^a^	44.71 ± 0.95 ^a^	53.93 ± 2.09 ^a^	66.56 ± 1.46 ^a^
BFB	*Salmonella enterica*	54.56 ± 2.01 ^c^	64.71 ± 0.98 ^c^	76.43 ± 1.70 ^c^	85.96 ± 1.78 ^c^
Beetroot					
G^+^	*Listeria monocytogenes*	67.37 ± 1.70 ^a^	55.33 ± 1.29 ^a^	45.14 ± 1.53 ^a^	36.44 ± 2.28 ^a,b^
	*Micrococcus luteus*	66.77 ± 1.24 ^a^	56.34 ± 1.82 ^a^	44.71 ± 1.06 ^a^	35.40 ± 1.37 ^a^
	*Staphylococcus aureus*	75.98 ± 2.56 ^b^	66.48 ± 2.23 ^b^	55.11 ± 2.08 ^b^	42.55 ± 1.20 ^b,c^
G^−^	*Enterobacter aerogenes*	77.63 ± 1.89 ^b^	56.34 ± 1.82 ^a^	46.30 ± 2.13 ^a^	35.04 ± 2.64 ^a^
	*Escherichia coli*	75.41 ± 1.16 ^b^	66.55 ± 2.90 ^b^	55.63 ± 1.24 ^b^	45.29 ± 1.91 ^c^
	*Yersinia enterocolitica*	77.26 ± 2.34 ^b^	65.95 ± 2.80 ^b^	55.49 ± 2.71 ^b^	46.10 ± 2.55 ^c^
Yeast	*Candida albicans*	75.78 ± 1.64 ^b^	66.21 ± 2.58 ^b^	56.40 ± 2.78 ^b^	44.77 ± 1.75 ^c^
	*Candida glabrata*	74.52 ± 1.23 ^b^	64.12 ± 1.13 ^b^	54.44 ± 1.56 ^b^	36.36 ± 2.03 ^a,b^
	*Candida krusei*	66.96 ± 2.18 ^a^	57.17 ± 0.62 ^a^	44.70 ± 1.00 ^a^	34.92 ± 2.26 ^a^
	*Candida tropicalis*	67.33 ± 2.53 ^a^	53.80 ± 0.76 ^a^	44.04 ± 1.48 ^a^	35.00 ± 2.69 ^a^
BFB	*Salmonella enterica*	76.73 ± 2.21 ^b^	67.29 ± 1.67 ^b^	56.62 ± 2.22 ^b^	45.70 ± 1.96 ^c^

Data are the mean (±SD) of three repetitions. Different letters in each column refer to significant differences (Tukey, *p* ≤ 0.05).

## Data Availability

Data will be made available upon request.

## Supplementary Materials

The following supporting information can be downloaded at: https://www.mdpi.com/article/10.3390/plants14132071/s1, Figure S1: GC/MS chromatogram of *Abies Alba* EO; Figure S2: Mass spectrum limonene; and Figure S3: Mass spectrum α-pinene.
