# Supplementary material for: Antimicrobial Effects of Abies alba Essential Oil and Its Application in Food Preservation
Source: Plants (Basel). 2025 Jul 7;14(13):2071. doi: 10.3390/plants14132071 (PMC12251771; doi:10.3390/plants14132071)
Supplement: Supplementary file 1 [file plants-14-02071-s001.zip › plants-3709227-supplementary.pdf]

Supplementary Materials

For

Antimicrobial effects of *Abies alba* Essential Oil and Its Application in Food Preservation

Milena D. Vukić[1*], Nenad L. Vuković[1], Marina Radović Jakovljević[2], Marija S. Ristić[1], Miroslava Kačániová[3,4]

[1] Department of Chemistry, Faculty of Science, University of Kragujevac, 34000 Kragujevac, Serbia; milena.vukic@pmf.kg.ac.rs (M.D.V.), nvchem@yahoo.com (N.L.V.), marija.jeremic@pmf.kg.ac.rs (M.S.R.)
[2] Department of Biology and Ecology, Faculty of Science, University of Kragujevac, 34000 Kragujevac, Serbia; marina.jakovljevic@pmf.kg.ac.rs (M.R.J.)
[3] Institute of Horticulture, Faculty of Horticulture and Landscape Engineering, Slovak University of Agriculture, Trieda Andreja Hlinku 2, 94976 Nitra, Slovakia; miroslava.kacaniova@gmail.com (M.K.)
[4] School of Medical & Health Sciences, University of Economics and Human Sciences in Warsaw, Okopowa 59, 01043 Warszawa, Poland; m.kacaniova@vizja.pl (M.K.)

Content

Figure S1. GC/MS chromatogram of *Abies Alba* EO

Figure S2. Mass spectrum limonene.

Figure S3. Mass spectrum α-pinene.

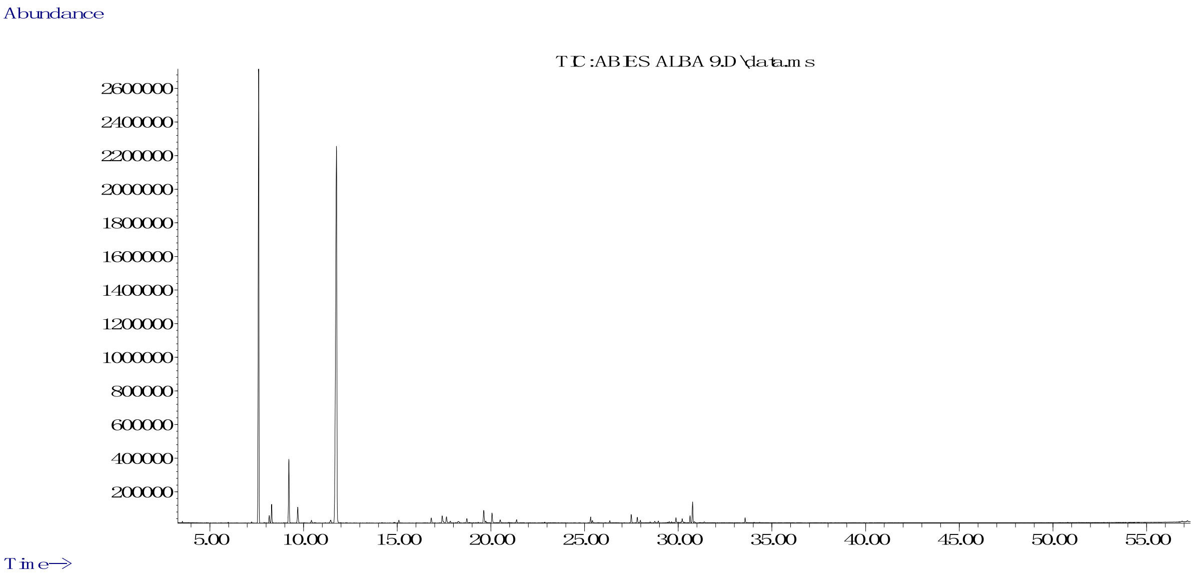

Figure S1. GC/MS chromatogram of *Abies Alba* EO

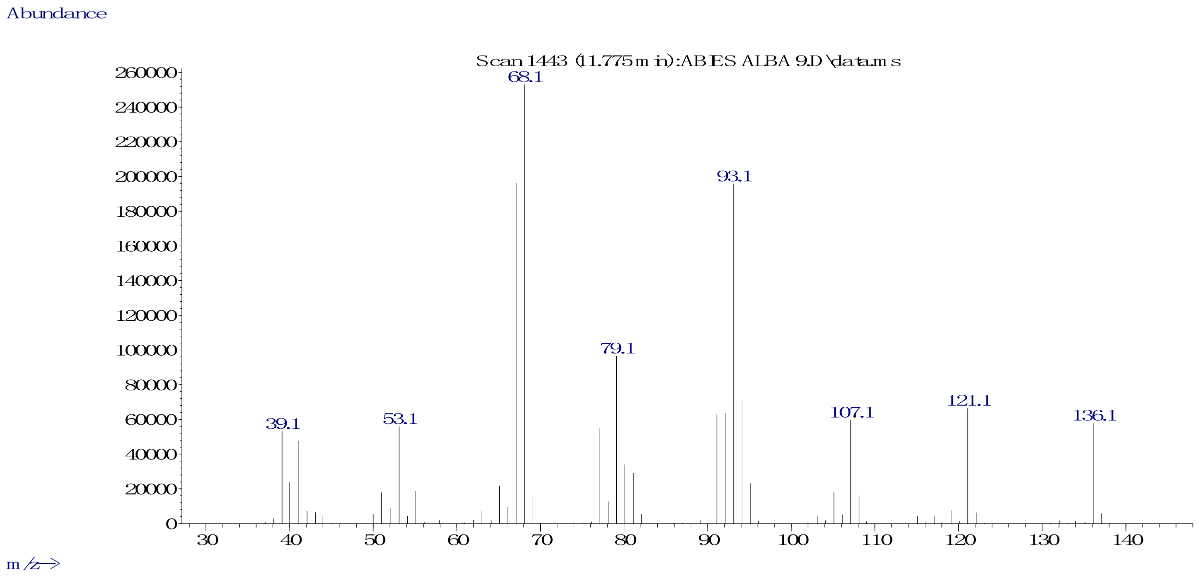

Figure S2. Mass spectrum limonene.

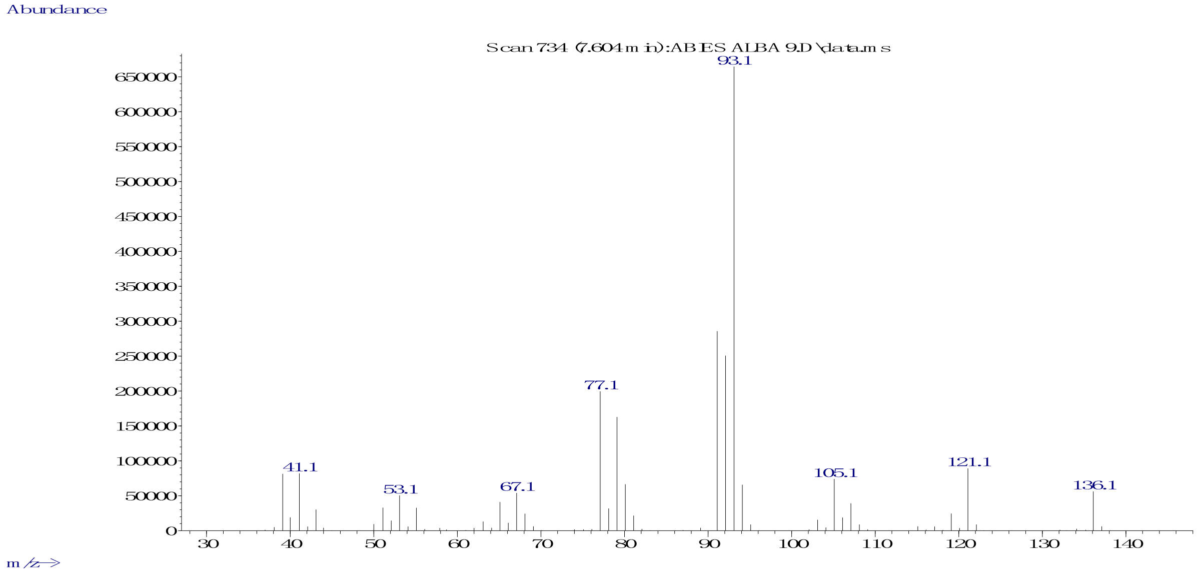

Figure S3. Mass spectrum α-pinene.
